# A systematic review of health promoting effects of consumption of whey-based fermented products on adults

**DOI:** 10.3389/fnut.2025.1651365

**Published:** 2025-08-20

**Authors:** Taner Sar, Bojana Bogovic Matijasic, Bojana Danilovic, Amparo Gamero, Mónica Gandía, Gabriela Krausova, Cristina Martínez-Villaluenga, Elena Peñas, Erfan Bagherzadehsurbagh, Özge Cemali, Dushica Santa, Ibrahim Ender Künili, Harun Kesenkas, Christophe Chassard, Smilja Pracer, Guy Vergères, Burcu Gündüz Ergün

**Affiliations:** ^1^Swedish Centre for Resource Recovery, University of Borås, Borås, Sweden; ^2^University of Ljubljana, Biotechnical Faculty, Department of Animal Science, Domzale, Slovenia; ^3^Faculty of Technology, University of Nis, Leskovac, Serbia; ^4^Faculty of Pharmacy and Food Sciences, Universitat de València, Burjassot, Valencia, Spain; ^5^Dairy Research Institute, Prague, Czechia; ^6^Institute of Food Science, Technology and Nutrition (ICTAN-CSIC), Madrid, Spain; ^7^Institute of Natural and Applied Sciences, Akdeniz University, Antalya, Türkiye; ^8^Department of Nutrition and Dietetics, Trakya University, Edirne, Türkiye; ^9^Faculty of Agricultural Sciences and Food - Skopje, Ss Cyril and Methodius University in Skopje, Skopje, North Macedonia; ^10^Department of Fishing and Fish Processing Technology, Faculty of Marine Science and Technology, Çanakkale Onsekiz Mart University, Çanakkale, Türkiye; ^11^Department of Dairy Technology, Ege University, Izmir, Türkiye; ^12^UCA, INRAE, VetAgro Sup, UMRF, Aurillac, France; ^13^Institute for Biological Research Sinisa Stankovic, National Institute of Republic of Serbia, University of Belgrade, Belgrade, Serbia; ^14^Research Division Microbial Food Systems, Agroscope, Berne, Switzerland; ^15^Health Biotechnology Centre of Excellence for Joint Research and Application (SABIOTEK), Yildiz Technical University, Istanbul, Türkiye; ^16^Department of Molecular Biology and Genetics, Faculty of Arts and Sciences, Yildiz Technical University, Istanbul, Türkiye

**Keywords:** fermented whey, functional foods, probiotics, bioactive peptides, immune modulation, cardiovascular health, gastrointestinal health, randomized controlled trials

## Abstract

**Introduction:**

Fermented whey-based products show significant potential as functional foods, owing to their rich nutritional profile and the generation of bioactive compounds during fermentation. This systematic narrative review evaluates the health effects of fermented-whey consumption based on evidence from human studies in adults.

**Methods:**

A systematic literature search was conducted using electronic databases including, PubMed, Scopus, and Cochrane Library for studies published between 1.1.1970 and 31.12.2024. All human clinical studies conducted with adults over 18 years old were included in this study. Inclusion criteria were randomized controlled trials and clinical studies involving adults consuming fermented whey products. Data extraction and quality assessment were performed using CADIMA software and standardized protocols. Studies identified by the search strategy and extracted data were screened independently by 2 reviewers using the CADIMA software. Risk of bias was assessed using the Risk of Bias 2 tool.

**Results:**

After screening 1852 titles and abstracts and assessing 20 articles for eligibility, a total of 12 human intervention studies met the inclusion criteria and were included in the systematic narrative review. Consumption of fermented whey products was associated with improvements in muscle mass, glycemic control, lipid profiles (notably triglycerides and LDL cholesterol), immune function (e.g., increased natural killer cell activity), and reductions in oxidative stress and inflammation. Some studies also reported benefits for gastrointestinal and urinary tract health. The health effects were attributed to increased bioavailability of branched-chain amino acids, bioactive peptides, and microbial metabolites such as exopolysaccharides and short-chain fatty acids. Most interventions were well tolerated, with no serious adverse effects reported.

**Conclusion:**

Fermented whey products demonstrate promising health benefits across multiple physiological systems. While current evidence supports their use as functional food ingredients, further large-scale, long-term clinical trials are needed to confirm efficacy and elucidate mechanisms of action. Fermented whey appears to be a safe and versatile option for enhancing adult nutrition and health.

## Introduction

Whey, the principal by-product of dairy production, is generated in large quantities worldwide, with estimates ranging from 203 to 241 million tons by 2030 (1). Traditionally considered a by-product, whey is now recognized as a nutritionally rich material as it contains lactose, whey proteins, free amino acids, and minerals, thereby offering significant potential for sustainable valorization. One promising strategy to reduce whey’s environmental impact while producing high value-added products is its bioconversion through microbial fermentation, which uses whey as a growth substrate.

Depending on the type of dairy product manufactured, whey can be classified as sweet whey, which is obtained through enzymatic coagulation in cheese production, and acid whey, which is produced by acid coagulation of milk using starter cultures or added organic acids (2). Acid whey is predominantly generated during the production of Greek yoghurt, Greek-style yoghurt, and ‘fresh curd cheese’, where naturally produced or added acids also play key technological role. Commonly, acid whey is already a fermented product, as starter cultures are used in its production, either those for yoghurt such as *Streptococcus thermophilus* and *Lactiplantibacillus delbrueckii* subsp. *bulgaricus*, or those used for sour milk and fresh curd cheese, where *Lactococcus lactis* predominates. However, both sweet and acid whey can still serve as substrates for the growth of lactic acid bacteria (LAB), bifidobacteria, and other microorganisms capable of producing diverse metabolites. This process not only reduces the high biological oxygen demand and chemical oxygen demand of whey (3), but also generates compounds with potential health benefits, such as lactic acid, antimicrobial peptides, exopolysaccharides, short-chain fatty acids (SCFAs), enzymes, and more (4).

Fermented whey products have attracted growing interest due to their health-promoting properties, including immunomodulatory, antioxidant, and gut-supportive effects. These benefits are linked to a range of bioactive compounds naturally present in whey and those formed during fermentation (5). Regular consumption has been associated with improved gastrointestinal health, metabolic and immune response regulation, and the reduction of cardiovascular risk factors. These effects are largely attributed to the presence of live probiotic microorganisms, non-viable cells, their cellular components, and their metabolites (6–8). In this context, fermented whey represents a promising carrier for delivering both probiotic and postbiotic substances (9).

Furthermore, advances in fermentation technology and microbial biotechnology have enabled more targeted production of health-promoting compounds in fermented whey. The use of strain-specific cultures, co-fermentation techniques, and precision fermentation approaches has improved the yield and functionality of bioactive metabolites (10). These innovations have expanded the potential applications of whey-based products across both functional food and nutraceutical sectors.

Despite encouraging findings, the evidence supporting the health benefits of fermented whey remains fragmented. Although promising bioactivities have been reported, rigorously designed, placebo-controlled trials are still limited. A systematic evaluation of such studies is considered essential to identify research gaps, draw conclusions reliable for future research and promote innovation on fermented whey product development. Accordingly, this systematic review is conducted addressing the research question defined in the frame of “PIMENTO WG3,” thematic area “Satellite project S9. Food byproducts” (11): “What are the health effect of fermented whey?.” This review aims to consolidate existing knowledge on the health effects of fermented whey consumption in adults, with a focus on placebo-controlled clinical trials.

Specifically, the present systematic review aims to provide a comprehensive overview of existing placebo-controlled clinical trials in adults assessing the health effects of fermented whey products. The review examines health outcomes of fermented whey across various physiological systems, including cardiovascular, immune, gastrointestinal, urinary, nervous, and skeletal health, as well as blood glucose regulation. Reported outcomes are evaluated to determine the extent to which fermented whey products contribute to measurable health improvements and whether they offer advantages over non-fermented dairy or placebo interventions. Particular attention is given to distinguishing fermented whey from unfermented products with added probiotics, and to analysing the bioactive compounds involved, their potential mechanisms of action, and their bioavailability. In doing so, this review also identifies current knowledge gaps and outlines future directions for innovation and research in this promising field.

## Methods

### Study protocol

The protocol for this study was developed as part of a broader project involving sixteen other studies focused on the ‘Health benefits and risks of fermented foods’ (11), conducted within the framework of the COST Action (COST CA20128) PIMENTO (Promoting Innovation of ferMENTed fOods). The finalized protocol was subsequently registered on the Open Science Framework (OSF) and is available at https://osf.io/evgqn/.

### Literature search

A comprehensive literature search was conducted using the electronic databases PubMed, Scopus, and the Cochrane Library for studies published from January 1, 1970, to December 31, 2024. The primary search term used was “whey,” with additional search criteria and the full search strategy detailed in Supplementary Tables 1–3. The search strategy was adapted from the Working Protocol developed by Todorovic et al. (11) within the PIMENTO COST Action and was specifically revised for this review using the keyword ‘fermented whey’, as outlined in the Working Protocol – Satellite 9 (S9), version dated October 3, 2024.

### Selection criteria

All clinical studies involving adults aged 18 years and older, whether healthy or diagnosed with a specific condition, were included in this review. Studies involving participants under 18 years of age, as well as *in-vitro* and animal trials, were excluded. However, some animal and *in-vitro* studies demonstrating health effects were also noted as supporting evidence. The intervention or exposure had to involve whey-based fermented products; studies using unfermented whey (i.e., whey not processed to fermentation after cheese production, such as without the addition of starter cultures) were not considered. Only studies published in English focusing on fermented whey were deemed eligible. Review articles, short reports, letters, comments, conference abstracts, and studies lacking sufficient information were excluded.

### Data extraction

Study selection and data extraction were carried out in accordance with the methodology outlined by (12). Reference selection was carried out using CADIMA software (13).

**Figure 1:**
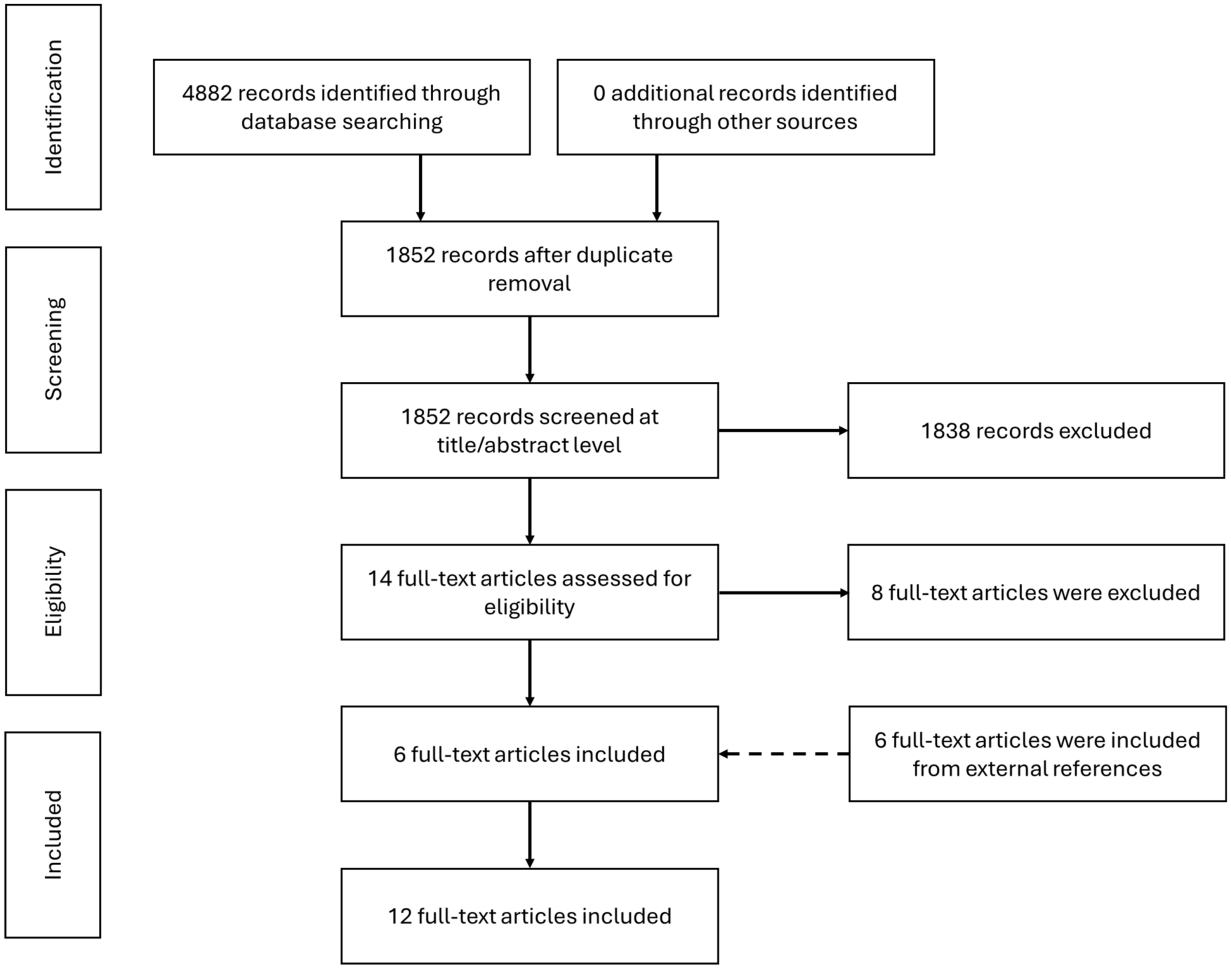
Flow diagram showing the study selection process and results of the systematic literature search.

A total of 4,882 studies (Figure 1) were identified that met the search strategy outlined in three electronic databases (PubMed, Scopus, and the Cochrane Library) and in Supplementary Tables 1–3. After removing duplicates and non-English articles, the remaining studies were independently screened at the title and abstract level by two reviewers using CADIMA.

14 articles that met the study’s inclusion criteria were then reviewed in full text by two reviewers. Additionally, to ensure comprehensive coverage, the reference lists of relevant review and systematic review articles, as well as the 14 included full-text articles, were researched to identify any potentially eligible studies not captured by the initial search strategy.

A flow diagram summarizing the number of articles identified, screened, and included is presented in Figure 1. Characteristics of the fermented whey products used in the included studies are summarized in Table 1. Data was extracted using a standardized form to ensure consistency and completeness across studies. For each included trial, information was collected on the study design and duration, sample size, and population characteristics. Detailed descriptions of the fermented whey intervention were recorded, including the type of product used, dosage, and duration of administration. Information on the comparator group—whether placebo, non-fermented dairy product, or another control—was also documented. Finally, the clinical outcomes assessed and the main findings reported by the authors were systematically extracted.

**Table 1:** Characterization of fermented whey products.

Fermented food	Raw material/substrate	Food geographical origin	Food form (dry/semiliquid/ liquid)	Used culture /cultures	Type of fermentation	Process	Main metabolites	References
Fermented whey product	Pasteurized and chilled whey from semi-hard cheese production		Liquid	*L. plantarum* MCC1 (DSM 23881) and *L. gasseri* MCC2 (DSM 23882)	Lactic fermentation	Pasteurized and chilled whey from semi-hard cheese production (cheese dust and fat removed, pH 6.4–6.6) was heated to fermentation temperature (37 °C ± 0.5 °C) and patented *L. plantarum* MCC1 (DSM 23881) and *L. gasseri* MCC2 (DSM 23882), strains with an ability to hydrolyze milk proteins, were added.		(21)
Fermented Maillard-reactive whey protein (F-MRP)	Whey protein concentrate	USA	Dry	*L. plantarum* LC01	Lactic fermentation			(8)
Fermented whey protein	Whey protein concentrates (WPC-80) and 3% glucose		Dry	*L. casei* DK211			Free amino acids	(16)
Malleable protein matrix (MPM)	Whey	Germany	Dry powder	*L. kefiranofaciens*	Lactic fermentation	Concentration of constituents with a centrifugation step following whey fermentation using *L. kefiranofaciens.*	Whey proteins such as lactoferrin and immunoglobulin, calcium or various fermentation metabolites (i.e., exopolysaccharides)	(15)
Malleable protein matrix (MPM) Yoghurt	Whey	Germany	Semiliquid	*L. kefiranofaciens*	Lactic fermentation	Fermentation of the whole whey was carried out according to the protocol proposed by Simard et al. (45), resulting in a malleable protein matrix (MPM).	Whey proteins, peptides, a proprietary strain of *L. kefiranofaciens*, exopolysaccharides, and Ca	(6)
Fermented vitamin E fortified whey beverage	Whey protein concentrate	Iran	Liquid	Yogurt starter (TY17A)	Lactic acid fermentation	After mixing the ingredients, the mixture was heated and pasteurized and fermentation was triggered by adding the starter and regulating the optimum temperature. For the vitamin E fortified beverage, vitamin E (600 IU as all-racemic-a-tocopherol) was added and homogenized throughout the mixture. That was followed by the addition of sterile mint flavor and final pasteurization.		(25)
Drinkable whey-based *L. acidophilus (johnsoni)* La1 culture supernatant	Enzymatic hydrolysate of demineralized whey proteins	Switzerland	Liquid	*L. acidophilus (johnsoni)* La1	Lactic acid fermentation	La1 was grown in a medium of the same composition as MRS, except that meat extract was replaced by an enzymatic hydrolysate of demineralized whey proteins (Nestlé Research Center, Orbe, Switzerland). The medium was heat sterilized (110 °C, 30 min) before being used for La1 culture. La1 spent culture supernatant was harvested by centrifugation, aromatized by an apple aroma, and stored at −20 °C.		(20)
Fermented whey drink	Whey	Finland	Liquid	*Lactobacillus* GG	Lactic fermentation			(18)
F-MRP (Fermented Maillard-Reactive Whey Protein)	Whey protein reacted with reducing sugars	Republic of Korea	Lyophilized powder	Not specified for fermentation; Maillard reaction products	Lactic acid fermentation	Reaction conditions optimized for Maillard reaction between whey protein and reducing sugars, followed by fermentation	Bioactive peptides (e.g., serine, leucine, histidine, arginine, sphinganine), Maillard reaction products, and sphingolipids	(7)
Milk Whey Culture with *P. freudenreichii* ET-3	Milk whey	Japan	Liquid or tablet (lyophilized form)	*P. freudenreichii ET-3*	Lactic acid fermentation	Optimized culture conditions using milk whey digested by protease Amano A for enhanced production of active compounds	1,4-Dihydroxy-2-naphthoic acid (DHNA), bifidogenic growth stimulators; increased butyrate concentration, recovery of *Lactiplantibacillus* and Enterobacteriaceae populations	(17)
Fermented whey	Deproteinised fermented organic whey (FWC) concentrate rich in L-(+)-Lactic acid (70 g/L)	Switzerland	Liquid	*Lactiplantibacillus* sp.	Lactic acid fermentation		L-(+)-Lactic acid (70 g/L)	(22)
*Lactobacillus* GG whey drink	Whey serves as the base substrate, along with apricot and peach for flavor.	Finland	Liquid	*Lactobacillus GG*	Lactic acid fermentation		Reduced metabolites associated with enzymatic activity: Decreased glycocholic acid hydrolase (reduced bile acid conversion to toxic secondary bile acids), urease (reduced ammonia production), *β*-glucuronidase (reduced production of toxicants and carcinogens), and tryptic activity	(19)

### Characteristics of fermented foods

Food characterization of the fermented whey products in the included clinical studies was based on several factors: the fermentation substrate, geographical origin, product form, type of microorganism used for fermentation, fermentation process, and key metabolite content. This information is summarized in Table 1.

### Quality and bias of human studies

In this systematic review, the quality and risk of bias of the included randomized controlled trials were assessed using the Cochrane Risk of Bias 2 (RoB 2.0) tool, which evaluates bias across five key domains: the randomization process, deviations from intended interventions, missing data, outcome measurement, and selection of reported results (14).

The risk of bias in non-randomized intervention studies was assessed using the ROBINS-I v2.0 tool (14), and studies were classified as having ‘low’, ‘moderate’, ‘serious’, or ‘critical’ risk of bias.

The assessment process was conducted with the support of members of the S9 working group, all of whom had received training in quality and bias assessment through online and in-person workshops organized by PIMENTO. Each study was independently assessed by at least two reviewers selected from within the group. Subsequently, all evaluations were reviewed by an experienced researcher. Discrepancies between reviewers were resolved by experienced researcher decision. This process aimed to enhance methodological consistency and strengthen the internal validity of the assessments. Conflicting evaluations of observational studies were addressed and resolved like Randomized Controlled Trials (RCTs).

### Supportive evidence – mechanism of action

The studies included in this systematic review are clinical trials conducted using fermented whey as mentioned above. *In-vitro* and animal studies, as well as other health-related research articles involving fermented whey, were not included in the main analysis. However, these studies were reviewed separately to provide supportive context for the health effects observed in human trials for better understanding the underlying mechanisms of action regarding the attributed health effects.

## Results and discussion

### Identification of pertinent human efficacy studies

#### Number of studies

A total of 12 human efficacy studies met the inclusion criteria and were included in the data analysis.

#### Type of study

The majority of the 12 studies employed randomized controlled trial (RCT) designs (7, 8, 15, 16). One study used open-label or crossover formats (17), which featured both short-term and long-term fermented whey supplementation periods.

Placebo-controlled crossover periods were incorporated in studies by (18, 19), focusing on short-term effects related to gastrointestinal health and mutagenicity. Michetti et al. (20) conducted a placebo-controlled, parallel-group study comparing the effects of fermented whey and omeprazole on *Helicobacter pylori.* Ausmees et al. (21) implemented a parallel design to evaluate urinary and inflammatory markers in men with lower urinary tract symptoms, while Smith et al. (22) used a pre-post design to evaluate changes in fecal-SCFAs profiles.

No observational studies (e.g., cohort, case–control, or cross-sectional) or summary publications (e.g., systematic reviews or meta-analyses) were included in this review. The predominance of RCTs, many of which used double-blind and placebo-controlled methodologies, strengthens the clinical evidence supporting the efficacy of whey-based fermented products.

#### Population

Three studies included healthy male and female participants (17, 18, 22), while one study involved only healthy male participants, and another included healthy individuals with mild to moderate facial acne vulgaris (23). Two studies enrolled non-diabetic (fasting serum glucose <126 mg/dL and 2-h serum glucose <200 mg d/L) and non-obese participants (18.5 kg m-2 ≤ BMI ≤ 30.0 kg m-2) (7, 8).

One study focused on elderly nursing home residents who reported defecation difficulties. Another included male and female participants with hypercholesterolemia, defined by LDL-C levels between 130 mg/dL and <190 mg/dL (24). A separate study enrolled individuals with metabolic syndrome, characterized by a waist circumference of 88–102 cm, triglycerides ≥150 mg/dL, HDL-C 40–50 mg/dL, systolic blood pressure ≥130 mmHg or diastolic blood pressure ≥85 mmHg, and fasting plasma glucose ≥100 mg/dL (6).

One study included only hemodialysis patients (25), another focused on patients infected with *H. pylori* (20), and one involved men with moderate lower urinary tract symptoms (21).

#### Intervention

The duration of intervention in the 12 selected studies ranged from 3 days to 3 months.

The fermented whey-based products used in these studies varied in form and composition. Interventions included fermented liquid whey products, *P. freudenreichii*-fermented whey cell-free supernatant, fermented whey protein, fermented Maillard-reactive whey protein (F-MRP), fermented multi-probiotic matrix, low-fat yogurt supplemented with whey-based multi-probiotic matrix, and a whey-based *L. acidophilus* (La 1) culture supernatant.

The administered doses varied across studies. For liquid products, doses ranged from 15 g twice daily to 100 mL and up to 660 mL per day. For solid or powdered formulations, the amount ranged from 2.67 g to 300 g.

#### Control

A wide variety of control interventions were used across the studies. In some cases, flavored water-based drinks were employed, such as a mixture of water with orange-peach syrup. In studies investigating F-MRP, maltodextrin served as the control. Other studies used unfermented versions of the test products, such as tablets or powders containing unfermented whey. Gelatin and hydrolyzed gelatin were also used as control substances in certain trials.

In one clinical trial, the control group received no intervention at all. In another, participants received pharmaceutical products like omeprazole or placebo pills resembling omeprazole. Some studies instructed control group participants to avoid the intake of probiotic-containing foods. In others, non-fermented whey protein or unfermented fruit-flavored placebo drinks were used. One study did not provide a description of the control product used.

#### Outcome

The reviewed studies investigated a wide range of health-related outcomes using both clinical measures and validated biochemical markers. These included areas such as muscle function, metabolic regulation, immune response, oxidative stress, urinary tract health, and gastrointestinal function. Although many studies reported beneficial effects following the consumption of fermented whey products, some yielded neutral results with no significant changes observed. Despite some variability in the specific outcomes assessed, the majority aligned with the expected physiological benefits attributed to fermented whey, such as supporting metabolic, immune, and digestive health.

### Characterisation of the food/constituent

#### Fermented whey

Whey protein, a by-product of cheese production, has been recognized for its nutritional profile and functional properties. Comprising 15–20% of total milk proteins, whey is particularly rich in essential branched-chain aminoacids (BCAAs) -such as leucine, isoleucine, valine, and cysteine- and functional bioactive components including *β*-lactoglobulin, *α*-lactoalbumin, glycomacropeptide, immunoglobulins, serum albumins, and lactoferrin (26). Most bioactive peptides in whey protein exist in inactive forms within the parent proteins, fermentation enhances their bioavailability by enabling microorganisms to secrete proteases that hydrolyze proteins and release bioactive peptides with specific health effects (16). Fermentation of whey proteins leads to the formation of angiotensin converting enzyme inhibitory (ACE-I) peptides that have anti-hypertensive activity (27). Also, many other bioactive molecules have been formed that possess antioxidant, immunomodulatory, and cholesterol lowering effects (Table 1). Various microbial strains have been employed in the fermentation of whey, including *Lactobacillus* and *Lactiplantibacillus* species (e.g., *Lactiplantibacillus plantarum*, *Lactobacillus gasseri*, *Lactobacillus casei*, *Lactobacillus kefiranofaciens*, *Lactobacillus acidophilus* [johnsonii], *Lactobacillus rhamnosus*, *Lactococcus lactis*, *Lactobacillus delbrieckii* subsp*. bulgaricus*, and *Lactobacillus paracasei* [syn. *Lacticaseibacillus casei*]), *Pediococcus pentosaceus*, *Bifidobacterium breve*, *Propionibacterium freudenreichii*, *S. thermophilus*, *Enterococcus malodoratus*, *E. faecium*, *Veillonella parvula*, and *Saccharomyces cerevisiae*. The health effects of the resulting fermented whey products have been investigated through both *in vitro* and *in vivo* studies and are discussed in this manuscript.

Fermented whey-based production processes have been demonstrated in various studies. For instance, a fermented whey beverage was developed using pasteurized and chilled whey from semi-hard cheese production, with cheese dust and fat removed (pH 6.4–6.6). The whey was heated to fermentation temperature (37 ± 0.5 °C) and inoculated with *L. plantarum* MCC1 (DSM 23881) and *L. gasseri* MCC2 (DSM 23882), both capable of hydrolyzing milk proteins (21). After 20 h of fermentation, the product was pasteurized. The pH dropped significantly from 6.14 to 3.99, and the fermented whey exhibited higher carbohydrate and dry matter content compared to the unfermented product. To improve sensory quality, orange-peach syrup was added to the final product. This fermented whey demonstrated health benefits, including improved urinary function, reduced lower urinary tract symptoms, decreased systemic oxidative stress markers, and improved seminal plasma inflammatory status (21).

A deproteinized, organic fermented whey concentrate (FWC) rich in L-(+)-lactic acid (70 g/L) was produced using a *Lactobacillus* species (22). FWC consumption significantly altered the fecal SCFAs profile, suggesting potential prebiotic activity. However, these findings require validation through controlled clinical trials. The effects may be attributed to the high lactic acid content, which could stimulate specific gut microbiota species.

*L. casei* DK211, isolated from Korean homemade kimchi, was used to ferment a whey protein fluid containing 7% whey protein concentrate (WPC-80) and 3% glucose (16). The mixture was dissolved in water (90%) and pasteurized at 60 °C for 30 min. The sterilized solution was then inoculated with DK211 (0.01% v/v, 5 × 10^10^ CFU/mL) and fermented at 38–40 °C for 30 h. Fermentation ended when the pH and acidity reached 4.3 and 1.0, respectively. The final product was dried with dextrin and rice powder using a freeze dryer. Fermentation resulted in substantial increases (>500%) in free amino acids such as alanine, proline, histidine, leucine, methionine, valine, glutamic acid, and asparagine. Total free amino acid content rose from 21.9 mg/100 g to 182.69 mg/100 g. Notably, the levels of BCAAs—leucine, valine, and isoleucine—increased significantly. While the protein content of WPC was 94%, the fermented whey protein had 70.41% protein in the molecular weight range of 5,000 Da or higher. An 8-week human study involving healthy male participants showed that fermented whey was more effective than unfermented WPC in promoting muscle mass development and enhancing dynamic balance performance (16).

#### Malleable protein matrix (MPM)

Malleable protein matrix (MPM) is classified as a whey protein hydrolysate by the Chemical Abstract Service (nr. 308,074–13-7). MPM is a unique whey-derived ingredient obtained through lactic acid fermentation by the species *L. kefiranofaciens* (6, 15, 24). The raw material can be concentrated by centrifugation prior fermentation or be used directly and the final product can be lyophilized or in a semiliquid or liquid form after powder reconstitution (6, 15, 24).

Diverse metabolites have been found in MPM and different health-related properties attributed to this ingredient. The main metabolites found in this fermented product involve increased concentration levels of whey proteins, such as lactoferrin and immunoglobulin; bioactive peptides, such as angiotensin-converting enzyme inhibitors; essential minerals, such as calcium and potassium; and exopolysaccharides (kefiran) synthesized during fermentation (6, 15, 24).

Numerous *in vivo* studies have demonstrated systemic anti-inflammatory and immunomodulating effects as well as triglyceride (TG) and LDL-C–lowering properties (6, 15, 24). In order to check if these beneficial properties also appear in humans, some clinical trials have been performed. In these trials, diverse metabolic markers have been measured after MPM consumption and are related to fat metabolism (total cholesterol, LDL and HDL cholesterol, triglycerides and lipoproteins), glucose metabolism (glucose, insulin, glycosylated hemoglobin) as well as anthropometric measurements (body weight, waist circumference) (6, 15).

Berthold et al. (15) carried out a randomized placebo-controlled trial including 161 subjects (50% male; age 54.5 ± 9.8 years, body mass index 26.3 ± 3.6 kg/m^2^) with hypercholesterolemia. After double-blind 12-weeks treatment with MPM, these authors observed 9.8% decrease in TG levels compared to placebo group. This reduction was much higher (20%) in the subset of subjects with starting TG levels at 150 mg/dL or above. In addition, LDL-cholesterol slightly decreased (1.5%) compared to placebo group and a positive effect was seen on hemoglobin. However, HDL-cholesterol, blood pressure, and fasting blood glucose remained unchanged (15). Gouni-Berthold et al. (6) carried out another randomized placebo-controlled trial including 187 patients with the metabolic syndrome. The patients were given twice per day low-fat yoghurt supplemented with MPM for 3 months and a significantly larger reduction (16%) of TG in comparison to placebo was observed in test group. This reduction was more pronounced (18%) in subjects with elevated fasting TG (≥ 200 mg/dL). Besides, benefits in other cardiovascular risk factors (plasma glucose, systolic blood pressure and body weight) were detected. In both clinical trials, MPM products were tolerated well without severe adverse events or just minor gastrointestinal effects (6, 15).

In conclusion, MPM has significant TG-lowering properties in subjects with combined hypercholesterolemia and higher TG levels as well as in patients with metabolic syndrome when administered together with low-fat yoghurt. MPM has also potential to improve multiple other cardiovascular risk factors and glucose metabolism, but further research needs to be done in this sense.

### Health benefits

The potential health effects of fermented whey found in the studies selected for this systematic review are shown in Table 2.

**Table 2:** The potential health effects of fermented whey products.

Whey based product	Population	Intervention		Control form	Outcomes reported	References
		Duration	Dose			
Fermented liquid whey product (*L. plantarum* MCC1 (DSM 23881) and *L. gasseri* MCC2 (DSM 23882))	46 men with moderate urinary tract symptoms	4 weeks	200 g/day of fermented liquid whey product	200 g/day of water and 12% w/w orange-peach syrup	The intake of a specially fermented whey product enhanced urinary function, alleviated lower urinary tract symptoms, and reduced systemic oxidative stress markers as well as inflammatory levels in seminal plasma.	(21)
Fermented Maillard-reactive whey protein (*L. plantarum* LC01)	80 Nondiabetic and Nonobese	8 weeks	6 g of the test product contained 4.2 g of F-MRP powder.	Same amount of the placebo product (maltodextrin)	The F-MRP group showed a significant increase in both body mass index (BMI) and NK cell activity. The F-MRP group exhibited a more reduction in LDL cholesterol compared to the placebo group.	(8)
Fermented whey (*P. freudenreichii* ET-3)	10 healthy male and four healthy female (24–41 years old)	1-week supplementation period + 4-week washout period + 1-week supplementation	Tablet contained of freeze dried *P. freudenreichii* ET-3 culture medium. 45 tablets per day (total 30 g culture medium)	placebo tablets (unfermented product)	There are no statistically significant differences between both supplementations for any measured parameter, and the incidence of gastrointestinal symptoms.	(17)
	11 healthy male (30–56 years old)	13 weeks	4 tablets per day (total 2.67 g culture medium)		Total protein, white blood cell count, hemoglobin, and mean corpuscular hemoglobin concentration significantly decreased from baseline, while mean corpuscular volume and urine pH increased. However, the authors mentioned that these changes were not attributed to *P. freudenreichii* ET-3 culture medium supplementation.	
Fermented Maillard-reactive whey protein	50 nonobese (man and woman)	8 weeks	NA	placebo	Fermented MRP significantly increased NK cell activity after 8 weeks across all assay conditions. IL-6 levels significantly decreased, while TNF-*α* levels notably increased within each group compared to baseline.	(7)
Fermented Malleable protein matrix (*L. kefiranofaciens*)	161 patients with hypercholesterolemia	6 weeks	2 × 15 g/d Fermented MPM	placebo (gelatin)	Fermented-MPM significantly reduced triglyceride (TG) levels in individuals with hypercholesterolemia, particularly in those with TG ≥ 150 mg/dL, surpassing the effects of the placebo. Given that 50% of participants showed a decrease in LDL-C.	(15)
Whey MPM yoghurt (Wheygurt)	197 Patients with metabolic sendrome	3 months	2 × 150 g Wheygurt per day (morning and evening)	placebo (hydrolysed gelatine)	Consuming low-fat yogurt supplemented with whey MPM twice daily for 3 months significantly reduces fasting TAG levels and improves multiple cardiovascular risk factors in patients with metabolic syndrome.	(6)
Fermented whey beverage	92 hemodialysis patients	8 weeks	3 × 220 mL of fermented whey beverage (15 g of whey protein concentrate)	control (no intervention)	Supplementation with whey protein and vitamin E through a new fermented whey beverage improved the nutritional status of hemodialysis (HD) patients in a short period, as reflected by better subjective global assessment scores and malnutrition-inflammation score.	(25)
Whey-based culture supernatant (*L. acidophilus* La1)	*H. pylori* infected volunteers (20; 8 Man and 12 Woman)	14 days	50 mL of whey-based culture supernatant (before meals and at bedtime)	omeprazole (20 mg) or omeprazole placebo matching pills	In 20 subjects (mean age 33.1 years), La1 supernatant treatment significantly reduced breath test values immediately, both in the omeprazole and placebo groups (*p* < 0.03). This reduction persisted for 6 weeks post-treatment. However, gastric biopsies confirmed the persistence of *H. pylori* infection.	(20)
*Lactobacillus* GG fermented whey drink	28 volunteers, mean age 39 years old (range 28–58)	3 days	*Lactobacillus* GG fermented whey drink 400 mL* (2 × 200 mL per day)	no dietary supplementation	There is no effect on fecal or urinart mutagenicity.	(18)
Fermented organic whey concentrate (FWC) drink rich in L-(+)-Lactic acid (70 g/L)	18 healthy (5 M + 13 W)	6 weeks	2 × 20 mL concentrated drink (every morning and evening)	NA	Daily consumption of a fermented whey product significantly altered the fecal SCFAs profile, suggesting potential prebiotic effects.	(22)
Fermented Whey Protein (*L. casei* DK211)	48 healthy middle-aged males	8 weeks	2 packs (37 g/pack) per day	control (non-fermented whey protein)	FWPS improved physical performance and muscle health, as shown by increased dynamic balance, grip strength (left), upper arm circumference, and flat leg circumference. These benefits were not observed in the WPCS group, suggesting that whey protein fermented by *L. casei* DK211 is an effective supplement for males engaged in regular resistance training	(16)
*Lactobacillus* GG fermented whey drink	Elderly Nursing Home Residents - 12 elder (78–91 age)	2 weeks baseline (placebo drink) + 2 weeks fermented whey drink + 2 weeks baseline (placebo drink)	2 × 1 dL of a *Lactobacillus*-GG- fermented whey drink (containing 108 cfu lactobacilli/ml) per day	placebo	The results suggest that a *Lactobacillus* GG-fermented whey drink alters bacterial metabolism but does not significantly affect bowel function.	(19)

#### Blood glucose, bone, and muscle health

Whey protein has been credited with numerous beneficial effects on human health, including its role in regulating blood glucose levels, maintaining bone mass, and improving nutritional level. Fermentation of whey protein using LAB further enhances its bioactive potential by promoting bone metabolism, regulating glucose concentrations, and influencing body mass index, among other effects.

The study carried out by Kim et al. (16) showed that fermentation of whey protein using a kimchi-specific LAB strain (*L. casei* DK211) increased the content of BCAAs such as leucine, valine, and isoleucine. These amino acids are beneficial for muscle biosynthesis and contribute to increased muscle mass and strength. Additionally, fermented whey was effective in promoting muscle mass development and maintaining dynamic balance when consumed by healthy adults for eight weeks. These results suggest that fermented whey protein could play a valuable role in improving musculoskeletal health in the general population.

Another beneficial effect was observed in a clinical trial with hemodialysis patients, which evaluated the effect of a fermented whey beverage on nutritional markers. Supplementation with whey protein, alone or in combination with vitamin E, gives positive results on nutritional parameters, such as the malnutrition-inflammation score. This improvement may be attributed to the antioxidant, anti-inflammatory, and anabolic effects of both whey protein and vitamin E. These findings support the potential use of enriched fermented whey drink as a complementary supplement in the management of malnutrition (25).

The Maillard reaction, a non-enzymatic browning process, leads to the formation of F-MRPs. Evidence suggests that supplementation with F-MRP whey protein may effect serum protein levels, helping to maintain or improve visceral proteins such as serum albumin, transferrin, and pre-albumin by modulating their concentrations (8). Additionally, due to its protective effects against lipid and protein oxidation, whey protein may also support muscle and weight gain, offering particular benefits for the elderly population (8).

Another functional product derived from whey protein fermentation is the MPM, which contains whey proteins, peptides, and exopolysaccharides. Animal studies have demonstrated its ability to modulate blood pressure and glucose levels (24). In human clinical trials involving patients with metabolic syndrome, MPM consumption resulted in reductions in body weight (approximately 1.5 kg over 3 months) and fasting plasma glucose levels by 10% (6). These glucose-lowering effects could be associated with the anti-inflammatory properties of MPM. Daily intake of natural MPM could, therefore, contribute to improvements in metabolic syndrome symptoms. Further positive effects were noted in a study involving individuals with hypercholesterolemia and normal triglyceride levels, where fasting glucose levels decreased slightly more in the MPM group compared to the control group, although the difference was not statistically significant (15).

In another study, healthy adults were supplemented with a product derived from serum fermentation by *P. freudenreichii* ET-3. During the supplementation period, blood glucose levels remained within normal ranges across all participants (17).

Collectively, these findings support the positive impact of fermented whey protein consumption, either as a standalone supplement or as an ingredient in fortified functional foods, on various health outcomes. The health benefits of fermented whey products are largely attributed not to the essential nutrient content but to the presence of bioactive compounds produced during fermentation, such as probiotic bacteria and microbial metabolites. In alignment with the European Food Safety Authority’s (EFSA) substantiation framework (28), robust evidence from human intervention studies is essential to support claims of enhanced immune function, maintenance of gastrointestinal health, improvement in urinary tract health, and defense against pathogenic microorganisms.

#### Immune function

Human studies consistently indicate that fermented whey-based products may support the strengthening of host immune defenses, particularly by enhancing innate immune responses. In two independent 8-week studies, the daily consumption of 6 g of fermented Maillard-reactive whey protein (F-MRP), produced using *Lactobacillus plantarum* LC01, significantly increased natural killer cell activity in healthy adults (7, 8). In Kang et al. (8) study, interleukin-6 (IL-6) concentrations were also significantly reduced from baseline, whereas tumor necrosis factor-*α* (TNF-α) levels increased within both intervention and control groups. As NK cells are essential components of the innate immune system, these findings support the role of F-MRP in enhancing early host defense mechanisms. Further evidence of immune-modulating potential was observed in an 8-week clinical trial involving hemodialysis patients receiving 220 mL of a fermented whey beverage (15 g protein concentrate) three times per week (25). In this study, supplementation with fermented whey beverage led to improvements in subjective global assessment and malnutrition-inflammation scores, suggesting reduced chronic inflammation and improved immune-nutritional status in this high-risk population.

#### Gastrointestinal health

Several studies have assessed the impact of fermented whey interventions on gastrointestinal microbiota and metabolic activity. In a 6-week intervention with healthy adults, daily consumption of a fermented organic whey concentrate rich in L-(+)-lactic acid (2 × 20 mL/day) significantly modified fecal short-chain fatty acids (SCFA) profiles, with notable increases in propionate and butyrate (22). These SCFAs are associatedimprovedgut barrier integrity and mucosal immunity.

Earlier trials focusing on elderly individuals have yielded mixed results. In a controlled study, *Lactobacillus GG*-fermented whey (2 × 100 mL/day) did not significantly alter bowel habits but led to colonization by the probiotic strain and a reduction in bacterial enzyme activity (e.g., tryptic and glycocholic acid hydrolase), potentially indicating a shift toward a healthier microbial metabolism (19). Likewise, in a short-term study of healthy adults, 3-day supplementation with 400 mL/day of *Lactobacillus GG*-fermented whey showed no effect on fecal or urinary mutagenicity (18). These findings suggest that while fermented whey products may influence microbial activity, such changes do not always result in immediate clinical outcomes.

The antimicrobial properties of fermented whey were further supported by a study in *H. pylori*-infected adults. A 14-day regimen of whey-based culture supernatant containing *L. acidophilus* La1 resulted in a significant, acid-independent reduction in breath test values, with effects persisting 6 weeks post-intervention, though eradication was not achieved (20). These outcomes point to a potential suppressive effect on *H. pylori*, possibly via microbial competition or antimicrobial metabolites.

#### Urinary health

The only study examining urinaryoutcomes evaluated supplementation of fermented whey beverage containing *L. plantarum* MCC1 and *L. gasseri* MCC2 in 46 men with moderate lower urinary tract symptoms (LUTS). Over a 4-week period, participants consumed 200 g/day of the fermented product in a randomized, double-blind, controlled trial (21). Compared to the placebo group, the intervention group experienced significant reductions in prostate-specific antigen, the oxidative stress marker 8-isoprostane (8-EPI), and seminal plasma interleukin-8 (IL-8) levels. These biochemical improvements were accompanied by clinically meaningful improvements in urinary symptom scores. The findings suggest that fermented whey products may exert anti-inflammatory and antioxidant effects that translate into improved urinary tract function, although longer trials are needed to assess the durability of these benefits.

#### Antioxidant activity and oxidative damage prevention

Daily consumption of 200 g of a whey product fermented with *L. plantarum* MCC1 and *L. gasseri* MCC2 for 4 weeks by men with moderate lower urinary tract symptoms resulted in a reduction of the systemic oxidative stress marker 8-isoprostanes (8-EPI) in urine compared to control group. The antioxidant properties of whey proteins are largely attributed to their high content in of BCCAs and cysteine, a precursor in glutathione synthesis (21).

#### Cardiovascular health

The effects of supplementation with *P. freudenreichii* ET-3 (strain 7,025), a cell-free product of whey fermentation by *P. freudenreichii* ET-3, on total and HDL cholesterol in healthy adults was evaluated by Uchida et al. (17). This was investigated through a randomized, double-blind, crossover trial (1 week, 3 g/day) and an open-label trial (13 weeks, 0.267 g/day). No significant changes in either cholesterol parameter were observed from baseline, nor were differences detected between placebo and active supplementation periods. Similarly, it was reported by Sohrabi et al. (25) that total, LDL, and HDL cholesterol levels were not altered in hemodialysis patients after consuming 220 mL of a whey beverage containing 15 g of whey protein concentrate, fermented by a yogurt starter culture (TY17A) with or without 600 IU vitamin E, three times per week for 8 weeks.

The supplementation with a MPM obtained through fermentation with *L. kefiranofaciens* (30 g/day) was evaluated in a multicenter, double-blind, randomized, placebo-controlled trial conducted over 6 weeks, with LDL-cholesterol levels as the primary outcome and triglycerides (TG) also assessed in subjects with hypercholesterolemia (15). A decrease of 2.1 ± 28.4% in TG levels was observed in the MPM group, with a 10% greater reduction from baseline compared to placebo. No significant changes in LDL-cholesterol were detected at the endpoint in the total cohort; however, a significant LDL-cholesterol reduction (5–10%) compared to placebo was noted at one recruitment center after 3 weeks (*p* = 0.084), 6 weeks (*p* = 0.016), and 12 weeks (*p* = 0.058). The TG-lowering effect was more pronounced in participants with baseline TG levels ≥150 mg/dL, reaching a 20% reduction compared to placebo (*p* = 0.040) after 12 weeks. In this subgroup, reductions in total cholesterol (−7.5%), LDL-cholesterol (−5.5%), and total cholesterol minus HDL cholesterol (−8.1%) were also recorded.

Subsequently, the same group investigated the effect of consuming two daily servings (150 g each) of a low-fat yogurt supplemented with MPM (Wheygurt™, containing 7 g of MPM per serving) on fasting triglyceride concentration (primary outcome), total cholesterol, LDL-cholesterol, and HDL-cholesterol in individuals with metabolic syndrome (6). A significantly greater reduction in fasting triglycerides (−16%, *p* = 0.004) was caused by MPM treatment compared to placebo, while no significant differences were found in other lipid or lipoprotein concentrations from baseline. The triglyceride reduction was especially notable in individuals with fasting triglyceride levels ≥200 mg/dL at baseline (−17.5%, *p* = 0.005).

Previous studies have demonstrated that whey proteins reduce *de novo* cholesterol synthesis in the liver of rats (29), inhibit the expression of genes involved in cholesterol and fatty acid synthesis and absorption (30), and increase fecal sterol excretion (29). However, the lipid-lowering effects observed with MPM in these studies are thought to result from the synergistic action of various product components, including whey proteins and peptides, exopolysaccharides, bacterial cell debris, and micronutrients (31). It should be emphasized that the lipid-lowering effect cannot be attributed to live lactobacilli, as the *Lactiplantibacillus* strain used in MPM production was thermally inactivated, consistent with findings from other studies (32, 33).

In a more recent randomized, double-blind, placebo-controlled study conducted over 8 weeks, supplementation with 6 g/day of a F-MRP powdered product (containing 4.2 g of F-MRP and fermented with *L. plantarum* LC01) was shown to reduce LDL-cholesterol levels (from 124.5 ± 5.27 to 118.3 ± 5.26 mg/dL) in individuals without diabetes or obesity. No changes in apolipoprotein B concentration were observed, indicating that alterations occurred in LDL particle size rather than particle number (8). It has been suggested that the reduction in LDL-cholesterol levels may be attributed to the inhibition of hepatic cholesterol synthesis, inhibition of intestinal cholesterol absorption by *β*-lactoglobulin, and/or reduced expression of genes involved in intestinal fatty acid and cholesterol absorption and synthesis.

### Quality and bias of human studies

In line with EFSA’s requirements for substantiating health claims, an outcome-related risk of bias assessment was conducted for each eligible study. Two non-randomized studies (19, 22) were evaluated using the ROBINS-I (Risk Of Bias In Non-randomized Studies of Interventions) tool (34).

The study by Smith et al. (22) was found to be of generally acceptable methodological quality. Although the absence of blinding, non-random participant selection, and incomplete data reporting introduced some methodological limitations, these issues were not deemed to invalidate the study’s main findings. Accordingly, this study was judged to carry a moderate risk of bias, and its results should be interpreted with appropriate caution. Further research with larger samples and randomized controlled designs is recommended to confirm the observed effects (22). In contrast, the study by Ling (19) exhibited substantial methodological shortcomings, including a small sample size, high attrition rate, absence of blinding, and lack of control for confounding variables. Due to these limitations, the study was deemed to have a serious risk of bias, and the generalizability of its findings remains highly restricted (19).

A total of ten parallel-design randomized controlled trials (excluding crossover and cross-sectional designs) were evaluated using the Cochrane Risk of Bias 2 (RoB 2) tool for assessing the risk of bias in randomized trials (14).

These studies were generally of strong methodological quality. Half of the randomized controlled trials minimized the risk of bias through appropriately conducted randomization procedures, double-blinding, and adequate allocation concealment (6, 15, 16, 20, 21).

However, in the remaining studies, variations in blinding procedures were observed, and either participants or intervention providers were not fully blinded (7, 8, 17, 25). This may have increased the risk of bias, particularly in studies using subjective outcome measures. Accordingly, the risk of bias was rated as “some concerns” in two studies (8, 18) and as “high risk” in three studies (7, 17, 20).

Seven of the evaluated studies were judged to have low risk of bias in terms of randomization, data collection, missing data, and outcome measurement.

The studies by Berthold et al. (15) and Gouni-Berthold et al. (6) include clinical trial registration numbers. In the other studies (7, 8, 16, 17, 20, 21, 25), ethical approval information is provided. Particularly, the presence of a clinical trial number contributes to transparency and is an important criterion in the assessment of bias.”

For the evaluation of intention-to-treat (ITT) analysis, a participant dropout rate of up to 5% is considered acceptable, as recommended by Cochrane (14). Only two studies clearly reported the use of ITT analysis to mitigate bias due to participant attrition (6, 15). In two studies, the proportion of missing data exceeded this threshold substantially, and no explanation was provided regarding the potential relationship between missingness and the outcomes (7, 17). Therefore, the risk of bias due to missing outcome data was considered significant.

Failure to perform ITT analysis implies the exclusion of participants who did not adhere to the intervention or withdrew from the study, which increases the risk of bias.

In conclusion, although the studies were conducted with a high level of methodological rigor, most demonstrated low risk of bias concerning randomization and missing data. However, the lack of pre-specified analysis plans and limitations in blinding procedures indicate that the findings should be interpreted with caution. Selection bias remains a significant concern in studies that do not ensure protocol transparency. The summary of risk of bias assessment is presented in Table 3.

**Table 3:** Risk of bias assessements for outcomes of interest across studies.

Study	Remarks	ROBİNS-I	Outcome	Domain 1: Bias arising from the randomization process	Domain S: Risk of bias arising from period and carryover effects^c^	Domain 2: Bias due to deviations from intended interventions	Domain 3: Bias due to missing outcome data	Domain 4: Bias in measurement of the outcome	Domain 5: Bias in selection of the reported result	Overall bias
Ausmees et al. (21)^a^	RCT parallel	NA	All outcomes	Low risk	NA	Low risk	Low risk	Low risk	Low risk	Low risk
Kang et al. (8)^a^	RCT parallel	NA	All outcomes	Low risk	NA	Some concerns	Low risk	Low risk	Low risk	Some concerns
Uchida et al. (17)^a^	RCT parallel	NA	All outcomes	Some concerns	NA	Low risk	Some concerns	Low risk	Some concerns	High Risk
Han et al. (7)^a^	RCT parallel	NA	All outcomes	Some concerns	NA	Low risk	Some concerns	Some concerns	Some concerns	High risk
Berthold et al. (15)^a^	RCT parallel	NA	All outcomes	Low risk	NA	Low risk	Low risk	Low risk	Low risk	Low risk
Gouni-Berthold et al. (6)^a^	RCT parallel	NA	All outcomes	Low risk	NA	Low risk	Low risk	Low risk	Low risk	Low risk
Sohrabi et al. (25)^a^	RCT parallel	NA	All outcomes	Low risk	NA	Some concerns	Low risk	Low risk	Low risk	Low risk
Michetti et al. (20)^a^	RCT parallel	NA	All outcomes	Low risk	NA	Some concerns	Low risk	Some concerns	Some concerns	High risk
Korpela et al. (18)^a^	RCT parallel	NA	All outcomes	Some concerns	NA	Some concerns	Low risk	Low risk	Low risk	Some concerns
Kim et al. (16)^a^	RCT parallel	NA	All outcomes	Low risk	NA	Low risk	Low risk	Low risk	Low risk	Low risk
Smith et al. (22)^b^	Non randomized intervention	Moderate risk	All outcomes	NA	NA	NA	NA	NA	NA	NA
Ling (19)^b^	Non randomized intervention	Critical risk	All outcomes	NA	NA	NA	NA	NA	NA	NA

^a^By default, all parallel group randomized controlled trials were appraised using the Risk of Bias 2 tool, following Sterne et al. (14), unless otherwise indicated.

^b^Non-randomized intervention studies were assessed using the ROBINS-I (following Sterne et al. (34) tool).

^c^Only randomized controlled crossover trials are subject to evaluation under this specific domain of the RoB 2 tool.

### Biological plausibility – mechanism of action

Fermented whey products can have antioxidants and anti-inflammatory properties due to the presence of whey proteins rich in BCAAs and cysteine. This can contribute to the synthesis of glutathione. Increased glutathione levels in men’s prostate epithelial cells protect cells from oxidative-induced death. This action of fermented whey products can improve the quality of life in men with lower urinary tract symptoms. Also, some peptides derived from whey proteins could decrease the infiltration of leukocytes and reduce the production of free radicals (21). Whey protein bio-converted by *L. casei* was found to effectively enhance physical performance, particularly in dynamic balance and muscle health. Fermentation of whey protein increases BCAAs and peptides, promoting muscle biosynthesis and anti-inflammatory effects (16). Daily intake of MRP whey protein fermented with *L. plantarum* LC01 could improve NK cell function, which showed a positive correlation with changes in interleukin-12 which were positively linked to variations in prealbumin. Moreover, F-MRP whey protein supplementation helps maintain or enhance visceral protein levels, including serum albumin, transferrin, prealbumin, and insulin-like growth factor-1. It also could result in a reduction in LDL cholesterol (8). Additionally, MPM demonstrated anti-inflammatory properties by inhibiting pro-inflammatory cytokines, including IL-1β, IL-6, and TNF-*α*, thereby supporting cardiovascular health (15). Also, whey MPM yogurt (whey yogurt) is shown to have various health effects. MPM reduces fasting triglyceride levels by enhancing lipoprotein lipase activity and decreasing hepatic VLDL synthesis. MPM improves insulin sensitivity and facilitates glucose uptake, leading to reductions in fasting glucose levels. MPM promotes vasodilation and vascular health by inhibiting ACE activity, resulting in lower systolic blood pressure. MPM reduces inflammation and oxidative stress through bioactive peptides derived from whey protein. A significant reduction in inflammatory markers such as hs-CRP was observed, supporting its anti-inflammatory benefits (6). In another study it has been demonstrated that MPM could regulate the activity of lipoprotein lipase which would lower plasma TG. Administration of whey proteins produces a significant reduction in dipeptidyl peptidase IV (DPP-IV) activity in the proximal small bowel that leads to an increase in levels of incretin, which inhibits glucagon release (which increases the blood glucose). More importantly MPM increases insulin secretion and decreases gastric emptying. Also, whey digestion generates di- and tripeptides that could act as DPP-IV competitive inhibitors like the well-known diprotin A (Ile-Pro-Ile) and diprotin B (Val-Pro-Leu). MPM probably has bioactive peptides that will inhibit DPP-IV activity before the luminal digestion and thus regulating blood glucose and insulin levels (24).

Microorganisms used for the fermentation of whey-based products can have multiple benefits for human health as they promote the growth of good bacteria in the body. Studies have demonstrated that the hypocholesterolemic effect of whey-fermented products can be enhanced by the synergistic effect between *L. casei* TMC0409, *S. thermophilus* TMC1543, and whey protein concentrate. LAB bind cholesterol, reducing its intestinal absorption, while the polysaccharides they produce may further inhibit steroid absorption in the intestine (35). The LAB strains used in whey fermentation exhibit antidiabetic and antioxidant properties, enhance biological activity through increased inhibition of *α*-amylase and *α*-glucosidase, and improve antioxidant activity. *L. casei* strains can be associated with the presence of antihypertensive peptides, while *L. acidophilus* and *Bifidobacterium* strains can cause higher antidiabetic activity in the fermented food due to greater α-glucosidase inhibition. Additionally, LAB strains during whey fermentation contribute to improvements in the high saturated fatty acid hypercholesterolemic index and produce bioactive peptides with ACE-inhibitory, antimicrobial, immunomodulatory, and antioxidant effects (27, 36). Production of organic acids, bacteriocin-like compounds and biofilm by *P. pentosaceus* and *L. plantarum* strains in liquid whey fermentation contribute antimicrobial activity, while the results of protein degradation contribute to the antioxidant properties and angiotensin-converting enzyme (ACE) inhibitory activity of fermented whey. They also have been demonstrated to bind heterocyclic aromatic amines and degrade nitrosamines and some types of biogenic amines. Peptides produced by the proteolytic activity of LAB starters are responsible for the antioxidant and ACE inhibitory activity of fermented whey (37). Additionally, studies have shown that specific strains of *L. acidophilus* produce inhibitory compounds (e.g., lactic and pyruvic acids) that are partially effective against *H. pylori* both *in vitro* and *in vivo*. This may help suppress *H. pylori* infection in humans (20). Whey beverage fermented with yoghurt culture and fortified with vitamin E can improve the nutritional status of hemodialysis patients. This improvement may be attributed to the antioxidant, anti-inflammatory, and anabolic properties of whey protein and branched-chain amino acid contents on stimulating protein synthesis, positive nitrogen balance, whey protein improving the anabolic effects of insulin (25). The analysis of the effects of pre-consumption of whey fermented with probiotic *L. rhamnosus* before ulcerative colitis induction revealed a significant reduction in disease activity and improvements in hematological parameters and histological scores. The notable decrease in pro-inflammatory markers and the increase in anti-inflammatory cytokine levels indicated a protective effect against colitis-induced inflammation (38). The fermented whey products enhance intestinal immunity by increasing secretory IgA levels, which help prevent pathogen adhesion to the intestinal mucosa (39). The application of whey fermented with *Lactobacillus GG* in elderly humans led to improved gut microbiota function. This was a result of reduced fecal glycocholic acid hydrolase and tryptic activity, which in turn decreased the formation of toxic secondary bile acids and putrefactive metabolites, contributing to better gut health (19). On the other hand, whey fermented with *Lactobacillus* GG had low or no impact on fecal and urinary mutagenicity or fecal enzyme activities induced by highly mutagenic protein pyrolysates in the diet (18). Regarding the immunity effects of fermented whey, it has been demonstrated that fermented whey could stimulate the innate immune response by increasing CD4 and CD8-positive T-cell counts and promote the elimination of *Listeria monocytogenes* in mice. Significant enhancement of the elimination of *L. monocytogenes* (bactericidal effect) from the spleen and liver was seen due to stimulation of the innate immune system - increased gene expression of IFN-gamma in the spleen, following the activation of macrophages which subsequently enhanced elimination of *L. monocytogenes* (40).

### Biological plausibility – bioavailability

The term “bioavailability” refers to the percentage of nutrients or functional ingredients that are absorbed, distributed to the tissues, metabolized, and eventually excreted by the body (41). There is scarce information in the literature regarding bioavailability of bioactive compounds present in fermented whey. Most of the studies focus on *in vitro* studies with fermented whey beverages where lactic acid fermentation improves the bioavailability of peptides, vitamins, minerals and even polyphenols (42, 43). Bacterial proteolytic activity results in the hydrolysis of proteins to small peptides and amino acids, which are easily absorbed, whereas LAB phytases hydrolyze phytic acid preventing mineral chelation (42). Phytic acid is present in cereals, which are commonly used as ingredients in whey beverage formulations (42). One *in-vitro* study employing Caco-2 cells compared the transepithelial transport and bioavailability of total polyphenols in fermented and unfermented milk whey, pointing out that lactic acid fermentation slightly increases those parameters up to 2.5% (43).

Finally, a randomized, non-blinded, and monocentric human pilot study including healthy volunteers between 18 and 30 years investigated the bioavailability of folates in biofortified fermented whey beverages. The biofortification was done with *Bifidobacterium* and *Streptococcus* strains, which naturally enhanced folate content (44). The results showed an average bioavailability of 17.1% in fermented whey (44). However, the statistical significance of this study was poor due to the low number of participants.

In this way, fermentation emerges as a promising tool to improve bioavailability of nutrients and bioactive compounds in whey but further studies need to be done in this sense.

### Characterization of the relationship between consumption of fermented whey and the functional effect

To characterize the relationship between consumption of the FF and the functional health effects, according to EFSA, it is important to consider a few points. If the study population has been sufficiently representative of the target population, the similarity of the study conditions to the conditions of free-living subjects, the sustainability of the health effect (s) over time with the continuous consumption of FF, minimum effective dose of the FF, the effective dose with which the study has been performed, and comparison of the FF intake in the study with the reasonable daily intake of the food.

In the studies performed by Ausmees et al. (21) the target population is men with urinary tract symptoms. The study has ensured a well-representing study population by thorough clinical examination. In the work by Berthold et al. (15), the study population is high on subject numbers with a well distribution in terms of age and gender, who have been selected after thorough clinical examination. The study population is a good representation of the target population who are adults with hypercholesterolemia. Similarly, Gouni-Berthold and colleagues selected suitable candidates representing their target population who are people with metabolic syndrome (6). In the study performed by Sohrabi et al. (25), the subjects were selected from the patients already receiving hemodialysis treatment which indicates the suitability of study population for the study regarding target population representation. Michetti et al. (20), also created a suitable and representative study population of people with *H. pylori* infection who were the target population of the study. In the studies performed by (7, 8, 18, 19, 22) the aim has been the study of the effects of their respective interventions on healthy populations, and they have selected healthy subjects, representing a healthy population. In the studies performed by Uchida et al. (17) the study population were healthy people, representing their target population, however, the low number of subjects and the high ratio of male subjects are not adequate representations of the target population which can potentially influence the study results. In the study performed by Kim et al. (16), although the target population is “middle-aged Korean adults,” all the selected subjects for the study population have been males which would cause the study population to not be a good representation of the target population.

Regarding the condition under which the effects have been achieved, all the studies except Sohrabi et al. (25) maintain consistent free-living conditions and the effects of the interventions were assessed under normal conditions. However, in the work of Sohrabi and colleagues the place of the consumption of the intervention was according to the patients. The patients were asked to consume the intervention product after their hemodialysis treatment either at home or at the clinic.

All studies except Uchida et al. (17) and Korpela et al. (18), demonstrated consistent and sustainable effects of the intervention during the continuous consumption of the product. Uchida et al. (17) found no significant effect regarding the consumption of the intervention during the two periods of the study. Similarly, in the study performed by Korpela et al. (18), no health effect was observed regarding the consumption of fermented whey-based intervention. However, these results do not include the long term effects of consuming the products either with continuous consumption or without. This gap should be addressed in future research. None of the studies defined the minimum effective dose of the intervention in their respective works. However, this is an important step in characterizing the effects of the interventions used in the studies. Addressing this gap will illuminate the path for many details regarding the relationship between the intervention fermented whey products and their health effects. However, the effective dose of intervention has been described in every study except for the study by Uchida et al. (17), which although has described the dose used in the study there has been no significant effect regarding the consumption of the intervention.

The intake amounts used in all the studies are in a reasonable range and can be consumed daily as part of a balanced diet except for the work of Uchida et al. (17) which in one of the studies performed the subjects have been asked to intake 45 tablets containing the bioactive compounds.

All the information regarding the characterization of the relationship between the consumption of fermented whey products and health effects has been summarized in Table 4.

**Table 4:** A summary of criteria for the characterization of the relationship between the fermented whey and health effects.

Fermented food	Study population representative of the target population	Intervention conditions	Effects sustainability	Minimum effective dose	The effective dose used in the study	Suitability for reasonable consumption	References
Fermented whey product	Yes	Free-living	The effects were sustained along with FF intake.	Not reported	200 g/day of fermented liquid whey product	Yes	(21)
Fermented Maillard-reactive whey protein (F-MRP)	Yes	Free-living	The effects were sustained along with FF intake.	Not reported	6 g of the test product contained 4.2 g of F-MRP powder.	Yes	(8)
Fermented whey protein	No. Low number of participants and high ratio of male subjects.	Free-living	The effects were sustained along with FF intake.	Not reported	2 packs (37 g/pack) per day	Yes	(16)
Malleable protein matrix (MPM)	Yes	Free-living	The effects were sustained along with FF intake.	Not reported	2 × 15 g/d Fermented MPM	Yes	(15)
Malleable protein matrix (MPM) Yoghurt	Yes	Free-living	The effects were sustained along with FF intake.	Not reported	2 × 150 g Wheygurt per day (morning and evening)	Yes	(6)
Fermented vitamin E fortified whey beverage	Yes	Some subjects might have digested the intervention product in a clinical condition.	The effects were sustained along with FF intake.	Not reported	3 × 220 mL of fermented whey beverage (15 g of whey protein concentrate)	Yes	(25)
Drinkable whey-based *L. acidophilus (johnsoni)* La1 culture supernatant	Yes	Free-living	The effects were sustained along with FF intake.	Not reported	50 mL of whey-based culture supernatant (before meals and at bedtime)	Yes	(20)
Fermented whey drink	Yes	Free-living	No significant effects were observed with the consumption of fermented whey.	Not reported	*Lactobacillus* GG fermented whey drink 400 mL* (2 × 200 mL per day)	Yes	(18)
F-MRP (Fermented Maillard-Reactive Whey Protein)	Yes	Free-living	The effects were sustained along with FF intake.	Not reported	Not reported	NA	(7)
Milk Whey Culture with *P. freudenreichii* ET-3	No. The subjects were all males which restricts the outcome, and it cannot be expanded to all adults.	Free-living	No significant effects were observed.	Not reported	Study 1: Tablet containing freeze dried *P. freudenreichii* ET-3 culture medium. 45 tablets per day (total 30 g culture medium) Study 2: 4 tablets per day (total 2.67 g culture medium)	No	(17)
Fermented whey	Yes	Free-living		Not reported	2 × 20 mL concentrated drink (every morning and evening)	Yes	(22)
*Lactobacillus* GG whey drink	Yes	Free-living		Not reported	2 × 1 dl of a *Lactobacillus*-GG- fermented whey drink (containing 108 cfu lactobacilli/ml) per day	Yes	(19)

Various health effects have been demonstrated to be a result of fermented whey in these studies. In general, the evidence reported in these studies is convincing about health effects of fermented whey products. However, deeper and more detailed investigations must be performed in future research. More information regarding the bioactive compounds present in fermented whey products, the dose/response relationship, minimum effective dose, and influence over time, could be illuminating in understanding the underlying mechanisms of these compounds and related health effects which consequently would facilitate their usage according to the health effects. This needs to be addressed in future research. Identifying the bioactive compounds would also put in light the synergistic effects between various compounds present in the product and might potentially open the path for novel treatment methods. Due to the complex food matrix and various biochemical changes following processing fermentation, the long-term effects of the consumption of fermented whey products should also be investigated in detail.

### Substantiation of a causal relationship between consumption of the fermented food and the functional effect

The data of relationship between consumption of FWP and the functional effect is summarized in Table 5. Most of the data reflects the research of only one study, as for the effect of FWP on reduced lower urinary tract symptoms, physical performance, nutritional status, *H. pylori* infection and gut microbiota composition (Table 5). Multiple studies have analyzed the application of FWP in the reduction of cholesterol levels. Although the research of Kang et al. (8) and Han et al. (7) is consistent in the results, both stated that further research is needed to confirm the increase in immunity by consumption of F-MRP. Similar was observed for MPM in the papers of Berthold et al. (15) and Gouni-Berthold et al. (6). Additionally, the study of Ausmees et al. (21) in the research of the effect of FWP on the urinary tract in men points out that there are not enough data to confirm the reliability of the data. None of the studies represented the dose–response relationship since all FWP products were administered in only one dose. This indicates a large gap in data availability for the determination of substantiable relationship between the consumption of FWP and health effect.

**Table 5:** A summary of the criteria for determination of relationship between the consumption of FWP and the functional effect.

Fermented food	Specificity of the effect	The dose–response relationship	Magnitude of the effect and physiological relevance	Consistency of the effect across studies	References
Fermented whey product	Reduced lower urinary tract symptoms	One dose	Effect is modest with clinical target range	Only one study is available	(21)
Fermented Maillard-reactive whey protein (F-MRP)	Increased immunity and reduced LDL-cholesterol	One dose	The effect is modest and requests further examination	Consistent with the study of Han et al. (7)	(8)
Fermented whey protein	Enhanced physical performance,	One dose	Strong effect, physiologically relevant.	Only one study is available	(16)
Malleable protein matrix (MPM)	Reduced LDL-cholesterol and TAG	One dose	Strong effect, clinically relevant	Consistent with the study Gouni-Berthold et al. (6)	(15)
Malleable protein matrix (MPM) Yoghurt	Reduced LDL-cholesterol and TAG	One dose	Strong effect, physiologically relevant	Consistent with the study Berthold et al. (15)	(6)
Fermented vitamin E fortified whey beverage	Improved nutritional status	One dose	Moderate effect in hemodialysis patients, physiologically relevant.	Only one study is available	(25)
Drinkable whey-based *L. acidophilus (johnsoni)* La1 culture supernatant	Suppressive effect in *H. pylori* infection	One dose	The effect is proven *in vitro* but not *in vivo*.	Only one study is available.	(20)
*Lactobacillus* GG fermented whey drink	Effect on fecal/urinary mutagenicity and fecal enzyme activities	One dose	No effect	Consistent with the study of Ling et al. (19)	(18)
F-MRP (Fermented Maillard-Reactive Whey Protein)	Enhancing of the immunity	One dose	The effect is modest and requests further examination	Consistent with the study of Kang et al. (8)	(7)
Milk Whey Culture with *P. freudenreichii* ET-3	Safety of high doses of *P. freudenreichii* ET-3 culture	One dose	Safe for use, no significant effect compared to placebo.	Only one study	(17)
Fermented whey	Gut microbiota composition	One dose	No control group so the effect cannot be assessed.	Only one study	(22)
*Lactobacillus* GG whey drink	Fecal enzymes	One dose	No effect	Consistent with the study of Korpela et al. (18)	(19)

### Safety

The safety profile of whey-based fermented products was evaluated across a variety of populations, including healthy adults, older individuals, and those with specific health conditions such as metabolic syndrome or renal disease. Overall, the interventions were well tolerated, with no serious or treatment-related adverse effects reported in the majority of studies. For example, Ausmees et al. (21) used a whey beverage fermented with *L. plantarum* MCC1 and *L. gasseri* MCC2 and observed no adverse effects in men with moderate lower urinary tract symptoms. Similarly, Michetti et al. (20) administered a whey-based culture supernatant of *L. acidophilus* La1 to adults with *H. pylori* infection and reported no safety concerns. In studies by Kim et al. (16) and Kawase et al. (35), fermented whey protein using *L. casei* strains was also well tolerated in healthy male adults. These findings suggest that fermentation with such strains does not introduce safety risks under the tested conditions.

In Gouni-Berthold et al. (6), the most commonly reported adverse events were gastrointestinal symptoms such as flatulence, diarrhea, nausea, and upper abdominal discomfort. These symptoms were mild, non-serious, and occurred at a similar rate in the placebo group, indicating good overall tolerability. Similarly, Uchida et al. (17) documented gastrointestinal complaints such as bloating, diarrhea, and nausea during high-dose supplementation with *P. freudenreichii* ET-3, but these were also seen during placebo and were not attributed to the intervention.

No specific populations were identified who should avoid fermented whey products. However, most studies excluded individuals with milk protein allergies, lactose intolerance, or chronic disease, implying that such groups should exercise caution or consult a healthcare provider before consumption.

None of the studies issued formal warnings regarding excessive intake. However, most trials were conducted in healthy or mildly affected individuals and excluded those with serious chronic conditions. As such, individuals with kidney disease or other metabolic disorders may require medical supervision before regular use.

## Limitations and summary of evidence

This systematic review highlights the expanding evidence base suggesting potential health benefits of fermented whey products. Clinical studies indicate positive effects on metabolic regulation, gastrointestinal and cardiovascular health, and immune function, likely mediated by fermentation-enhanced bioactive compounds such as peptides, SCFAs, exopolysaccharides, and other microbial metabolites.

However, the overall quality and consistency of the evidence remain limited. Many studies suffer from small sample sizes, brief intervention periods, and lack standardization in both the composition and dosage of the interventions. Methodological issues such as absence of randomization, insufficient blinding, and selective outcome reporting further weaken the reliability of findings. Additionally, the heterogeneity of fermented whey formulations and diverse study populations hinder the ability to generalize results.

To strengthen the evidence base, future research should employ more rigorous study designs—specifically well-powered, long-term randomized controlled trials with clearly defined protocols and outcome measures. Mechanistic investigations are also needed to better understand the biological pathways involved and to assess potential variability in individual responses.

This review itself is subject to certain limitations. Only English-language publications were included to align with the collaborative goals of the COST Action framework. Moreover, the Embase database—recommended by Cochrane for systematic reviews in medicine—was not searched due to logistical constraints, potentially limiting the comprehensiveness of the literature base.

Nonetheless, this review is not intended to serve as definitive evidence for the efficacy of fermented whey products. Rather, it provides a structured synthesis of current findings and aims to lay the groundwork for more conclusive future research in this area.

## Conclusion

Fermented whey products show promising benefits for metabolic, gastrointestinal, cardiovascular, and immune health, largely due to fermentation-derived bioactive compounds. Evidence from randomized controlled trials suggests that fermented whey may also contribute to improved muscle mass, better blood glucose and triglyceride regulation, enhanced immune function, stronger antioxidant defense, and support for urinary health. Some trials further indicate potential benefits in cardiovascular markers, particularly in at-risk individuals. However, current evidence is limited by small sample sizes, short study durations, lack of standardization, and methodological weaknesses. More rigorous, long-term randomized controlled trials and mechanistic studies are needed to confirm these effects and clarify biological pathways. This review provides a structured overview of existing data but emphasizes that findings remain preliminary.

## Data Availability

The original contributions presented in the study are included in the article/Supplementary material, further inquiries can be directed to the corresponding authors.
