# Supplementary material for: A systematic review of health promoting effects of consumption of whey-based fermented products on adults
Source: Front Nutr. 2025 Aug 20;12:1651365. doi: 10.3389/fnut.2025.1651365 (PMC12405291; doi:10.3389/fnut.2025.1651365)
Supplement: Supplementary file 1 [file Table_1.docx]

**Supplementary Material**

**A systematic review of health promoting effects of consumption of whey-based fermented products on adults**

Taner Sar^1,*^, Bojana Bogovic Matijasic^2^, Bojana Danilovic^3^, Amparo Gamero^4^, Mónica Gandía^4^, Gabriela Krausova^5^, Cristina Martínez-Villaluenga^6^, Elena Peñas^6^, Erfan Bagherzadehsurbagh^7^, Özge Cemali^8^, Dushica Santa^9^, Ibrahim Ender Künili^10^, Harun Kesenkas^11^, Smilja Todorovic^12^, Guy Vergères^13^, Christophe Chassard^14^, Burcu Gündüz Ergün^15,16*^

^1^ Swedish Centre for Resource Recovery, University of Borås, 501 90 Borås, Sweden.

^2^ University of Ljubljana, Biotechnical Faculty, Department of Animal Science, Groblje 3, 1230 Domzale, Slovenia.

^3^ Faculty of Technology, University of Nis, Bulevar oslobodjenja 124, 16000 Leskovac, Serbia.

^4^ Faculty of Pharmacy and Food Sciences, Universitat de València, Vicente Andrés Estellés no number, 46100 Burjassot, Valencia, Spain.

^5^ Dairy Research Institute, Ke dvoru 12a, 160 00 Prague, Czech Republic.

^6^ Institute of Food Science, Technology and Nutrition (ICTAN-CSIC), José Antonio Novais, 6, 28040 Madrid, Spain.

^7^ Institute of Natural and Applied Sciences, Akdeniz University, 07070, Antalya, Türkiye.

^8^ Nutrition and Dietetics Department, Trakya University, Edirne, Türkiye.

^9^ Faculty of Agricultural Sciences and Food - Skopje, Ss Cyril and Methodius University in Skopje, Republic of North Macedonia.

^10^ Department of Fishing and Fish Processing Technology, Faculty of Marine Science and Technology, Çanakkale Onsekiz Mart University, Türkiye

^11^ Department of Dairy Technology, Ege University, Izmir, Türkiye.

^12^ Institute for Biological Research Sinisa Stankovic, National Institute of Republic of Serbia, University of Belgrade, Belgrade, Serbia.

^13^ Research Division Microbial Food Systems, Agroscope, Berne, Switzerland.

^14^ Human Nutrition Unit, INRAE, Université Clermont-Auvergne, Clermont-Ferrand, France.

^15^ Health Biotechnology Centre of Excellence for Joint Research and Application (SABIOTEK), Yildiz Technical University, Istanbul, 34220 Türkiye.

^16^ Department of Molecular Biology and Genetics, Faculty of Arts and Sciences, Yildiz Technical University, Istanbul, 34220, Türkiye.

**Corresponding authors:**

T.Sar ([st.taner@gmail.com](mailto:st.taner@gmail.com)), B.G.Ergün ([burcu.ergun@yildiz.edu.tr](mailto:burcu.ergun@yildiz.edu.tr))

**Table S1.** Search strategy performed in the systematic review and the number of publications identified in the PubMed electronic database.

| **No.** | **Query** | **Number of publications** |
| --- | --- | --- |
| **#1** | whey[tiab] OR whey[MESH] | 12,024 |
| **#2** | "Diet"[Mesh] OR "Life Style"[Mesh] OR "Eating"[Mesh] OR "Feeding Behavior"[Mesh] OR ((food[tiab] OR macronutrient*[tiab] OR eating[tiab]) AND (intake*[tiab] OR habit*[tiab] OR behavior*[tiab] OR pattern*[tiab])) OR diet*[tiab] OR intake[tiab] OR ingestion[tiab] OR suppl*[tiab] OR consumption[tiab] OR meal*[tiab] OR nutrient*[tiab] OR nutrit*[tiab] | 2,394,644 |
| **#3** | #1 AND #2 | 4,778 |
| **#4** | Diet Surveys[Mesh] OR "Cohort Studies"[Mesh] OR cohort*[Tiab] OR prospective[Tiab] OR longitudinal[Tiab] | 3,284,588 |
| **#5** | Randomized Controlled Trial[Publication Type] OR Controlled Clinical Trial[Publication Type] OR Pragmatic Clinical Trial[Publication Type] OR Clinical Study[Publication Type] OR Adaptive Clinical Trial[Publication Type] OR Equivalence Trial[Publication Type] OR Clinical Trial[Publication Type] OR Clinical Trial, Phase I[Publication Type] OR Clinical Trial, Phase II[Publication Type] OR Clinical Trial, Phase III[Publication Type] OR Clinical Trial, Phase IV[Publication Type] OR Clinical Trial Protocol[Publication Type] OR multicenter study[Publication Type] OR "Clinical Studies as Topic"[Mesh] OR "Clinical Trials as Topic"[Mesh] OR "Clinical Trial Protocols as Topic"[Mesh] OR "Multicenter Studies as Topic"[Mesh] OR "Random Allocation"[Mesh] OR "Double-Blind Method"[Mesh] OR "Single-Blind Method"[Mesh] OR "Placebos"[Mesh:NoExp] OR "Control Groups"[Mesh] OR "Cross-Over Studies"[Mesh] OR random*[Title/Abstract] OR sham[Title/Abstract] OR placebo*[Title/Abstract] OR ((singl*[Title/Abstract] OR doubl*[Title/Abstract]) AND (blind*[Title/Abstract] OR dumm*[Title/Abstract] OR mask*[Title/Abstract])) OR ((tripl*[Title/Abstract] OR trebl*[Title/Abstract]) AND (blind*[Title/Abstract] OR dumm*[Title/Abstract] OR mask*[Title/Abstract])) OR "control study"[tiab:~3] OR "control studies"[tiab:~3] OR "control group"[tiab:~3] OR "control groups"[tiab:~3] OR "healthy volunteers"[tiab:~3] OR "control trial"[tiab:~3] OR "control trials"[tiab:~3] OR "controlled study"[tiab:~3] OR "controlled trial"[tiab:~3] OR "controlled studies"[tiab:~3] OR "controlled trials"[tiab:~3] OR "clinical study"[tiab:~3] OR "clinical studies"[tiab:~3] OR "clinical trial"[tiab:~3] OR "clinical trials"[tiab:~3] OR Nonrandom*[Title/Abstract] OR non random*[Title/Abstract] OR non-random*[Title/Abstract] OR quasi-random*[Title/Abstract] OR quasirandom*[Title/Abstract] OR "phase study"[tiab:~3] OR "phase studies"[tiab:~3] OR "phase trial"[tiab:~3] OR "phase trials"[tiab:~3] OR "crossover study"[tiab:~3] OR "crossover studies"[tiab:~3] OR "crossover trial"[tiab:~3] OR "crossover trials"[tiab:~3] OR "cross-over study"[tiab:~3] OR "cross-over studies"[tiab:~3] OR "cross-over trial"[tiab:~3] OR "cross-over trials"[tiab:~3] OR ((multicent*[tiab] OR multi-cent*[tiab] OR open label[tiab] OR open-label[tiab] OR equivalence[tiab] OR superiority[tiab] OR non-inferiority[tiab] OR noninferiority[tiab] OR quasiexperimental[tiab] OR quasi-experimental[tiab]) AND (study[tiab] OR studies[tiab] OR trial*[tiab])) OR allocated[tiab] OR pragmatic study[tiab] OR pragmatic studies[tiab] OR pragmatic trial*[tiab] OR practical trial*[tiab] | 3,926,928 |
| **#6** | "Epidemiologic Methods"[Mesh:NoExp] OR "Epidemiologic Studies"[Mesh] OR "Observational Studies as Topic"[Mesh] OR "Clinical Studies as Topic"[Mesh] OR "Single-Case Studies as Topic"[Mesh] OR "Organizational Case Studies"[Mesh] OR observational study[Publication Type] OR validation study[Publication Type] OR clinical study[Publication Type] OR case reports[Publication Type] OR "observational study"[tiab:~3] OR "observational studies"[tiab:~3] OR "observational design"[tiab:~3] OR "observational analysis"[tiab:~3] OR "observational analyses"[tiab:~3] OR ((cohort*[tiab] OR prospective[tiab] OR follow-up[tiab] OR longitudinal[tiab] OR long-term[tiab] OR retrospective[tiab]) AND (study[tiab] OR studies[tiab] OR design[tiab] OR analysis[tiab] OR analyses[tiab] OR data[tiab] OR review[tiab])) OR case control*[tiab] OR case comparison*[tiab] OR case-referent[tiab] OR "population study"[tiab:~3] OR "population studies"[tiab:~3] OR "population analysis"[tiab:~3] OR "population analyses"[tiab:~3] OR "descriptive study"[tiab:~3] OR "descriptive studies"[tiab:~3] OR "descriptive design"[tiab:~3] OR "descriptive analysis"[tiab:~3] OR "descriptive analyses"[tiab:~3] OR "multidimensional study"[tiab:~3] OR "multidimensional studies"[tiab:~3] OR "multidimensional design"[tiab:~3] OR "multidimensional analysis"[tiab:~3] OR "multidimensional analyses"[tiab:~3] OR "cross-sectional study"[tiab:~3] OR "cross-sectional studies"[tiab:~3] OR "cross-sectional design"[tiab:~3] OR "cross-sectional analysis"[tiab:~3] OR "cross-sectional analyses"[tiab:~3] OR "cross-sectional research"[tiab:~3] OR "cross-sectional survey"[tiab:~3] OR "cross-sectional findings"[tiab:~3] OR natural experiment*[tiab] OR quasi experiment*[tiab] OR "nonexperimental study"[tiab:~3] OR "nonexperimental studies"[tiab:~3] OR "nonexperimental design"[tiab:~3] OR "nonexperimental analysis"[tiab:~3] OR "nonexperimental analyses"[tiab:~3] OR "prevalence study"[tiab:~3] OR "prevalence studies"[tiab:~3] OR "prevalence analysis"[tiab:~3] OR "prevalence analyses"[tiab:~3] OR case series[tiab] OR "case report"[tiab:~3] OR "case reports"[tiab:~3] OR "case study"[tiab:~3] OR "case studies"[tiab:~3] OR "case histories"[tiab:~3] | 8,425,381 |
| **#7** | "systematic review" | 316,051 |
| **#8** | #4 OR #5 OR #6 OR #7 | 10,130,160 |
| **#9** | #3 AND #8 | 1,954 |
| **#10** | #9 NOT (("Child"[Mesh] OR "Infant"[Mesh] OR "Adolescent"[Mesh]) NOT "Adult"[Mesh]) | 1,685 |
| **#11** | #10 NOT (("Animals"[Mesh] OR "Animal Experimentation"[Mesh] OR "Models, Animal"[Mesh] OR "Vertebrates"[Mesh]) NOT ("Humans"[Mesh] OR "Human Experimentation"[Mesh])) | 1,318 |
| **#12** | #11 NOT ("Breast Feeding"[Majr] OR "Milk, Human"[Majr]) | 1,306 |
| **#13** | #12 AND (English[Filter]) | 1,286 |
| **#14** | #13 AND (("1970/01/01"[Date - Publication] : "2023/08/20"[Date - Publication])) | 1,280 |

**Table S2.** Search strategy performed in the systematic review and the number of publications identified in the Scopus electronic database.

| **#** | **Query** | **Number of publications** |
| --- | --- | --- |
| **#1** | TITLE-ABS-KEY (whey) | [29.421](https://www.scopus.com/search/history/results.uri?origin=searchhistory&shid=17) |
| **#2** | TITLE-ABS-KEY ( ( ( food OR *nutrient* OR eating OR nutrit* ) W/6 ( intake* OR habit* OR behavior* OR pattern* OR consumption OR suppl* OR ingestion ) ) OR diet* OR meal* ) | [1,859,515](https://www.scopus.com/search/history/results.uri?origin=searchhistory&shid=18) |
| **#3** | #1 AND #2 | [5.387](https://www.scopus.com/search/history/results.uri?origin=searchhistory&shid=19) |
| **#4** | TITLE-ABS-KEY (random* OR sham OR placebo* ) OR TITLE-ABS-KEY ((singl* OR doubl* ) W/1 (blind* OR dumm* OR mask* )) OR TITLE-ABS-KEY ((tripl* OR trebl* ) W/1 (blind* OR dumm* OR mask* )) OR TITLE-ABS-KEY (control* W/3 (study OR studies OR trial* OR group* )) OR TITLE-ABS-KEY (clinical W/3 (study OR studies OR trial* )) OR TITLE-ABS-KEY (Nonrandom* OR "non random*" OR non-random* OR quasi-random* OR quasirandom* ) OR TITLE-ABS-KEY (phase W/3 (study OR studies OR trial* )) OR TITLE-ABS-KEY ((crossover OR cross-over ) W/3 (study OR studies OR trial* )) OR TITLE-ABS-KEY ((multicent* OR multi-cent* ) W/3 (study OR studies OR trial* )) OR TITLE-ABS (allocated) OR TITLE-ABS-KEY (("open label" OR open-label ) W/5 (study OR studies OR trial* )) OR TITLE-ABS-KEY ((equivalence OR superiority OR non-inferiority OR noninferiority ) W/3 (study OR studies OR trial* )) OR TITLE-ABS-KEY ("pragmatic study" OR "pragmatic studies" ) OR TITLE-ABS-KEY ((pragmatic OR practical ) W/3 trial* ) OR TITLE-ABS-KEY ((quasiexperimental OR quasi-experimental ) W/3 (study OR studies OR trial* )) OR TITLE (trial) OR KEY (trial) | [13,867,178](https://www.scopus.com/search/history/results.uri?origin=searchhistory&shid=20) |
| **#5** | TITLE-ABS-KEY(observational W/3 (study OR studies OR design OR analysis OR analyses )) OR TITLE-ABS-KEY(cohort*) OR TITLE-ABS-KEY(prospective W/7 (study OR studies OR design OR analysis OR analyses )) OR TITLE-ABS-KEY(("follow up" OR followup ) W/7 (study OR studies OR design OR analysis OR analyses )) OR TITLE-ABS-KEY((longitudinal OR longterm OR (long W/1 term )) W/7 (study OR studies OR design OR analysis OR analyses OR data )) OR TITLE-ABS-KEY(retrospective W/7 (study OR studies OR design OR analysis OR analyses OR data OR review )) OR TITLE-ABS-KEY((case W/1 control ) OR (case W/1 comparison ) OR (case W/1 controlled )) OR TITLE-ABS-KEY(case-referent W/3 (study OR studies OR design OR analysis OR analyses )) OR TITLE-ABS-KEY(population W/3 (study OR studies OR analysis OR analyses )) OR TITLE-ABS-KEY(descriptive W/3 (study OR studies OR design OR analysis OR analyses )) OR TITLE-ABS-KEY((multidimensional OR (multi W/1 dimensional )) W/3 (study OR studies OR design OR analysis OR analyses )) OR TITLE-ABS-KEY(cross W/1 sectional W/7 (study OR studies OR design OR research OR analysis OR analyses OR survey OR findings )) OR TITLE-ABS-KEY((natural W/1 experiment ) OR (natural W/1 experiments )) OR TITLE-ABS-KEY(quasi W/1 (experiment OR experiments OR experimental )) OR TITLE-ABS-KEY(("non experiment" OR nonexperiment OR "non experimental" OR nonexperimental ) W/3 (study OR studies OR design OR analysis OR analyses )) OR TITLE-ABS-KEY(prevalence W/3 (study OR studies OR analysis OR analyses )) OR TITLE-ABS-KEY("case series") OR TITLE-ABS-KEY(case W/3 (report OR reports OR study OR studies OR histories )) | [9,436,269](https://www.scopus.com/search/history/results.uri?origin=searchhistory&shid=21) |
| **#6** | TITLE-ABS-KEY "systematic review" | [47](https://www.scopus.com/search/history/results.uri?origin=searchhistory&shid=22) |
| **#7** | #4 OR #5 OR #6 | [19,716,337](https://www.scopus.com/search/history/results.uri?origin=searchhistory&shid=23) |
| **#8** | #3 AND #7 | [2.728](https://www.scopus.com/search/history/results.uri?origin=searchhistory&shid=24) |
| **#9** | (KEY(animal* OR nonhuman)) AND NOT (KEY(human*)) | [6,665,269](https://www.scopus.com/search/history/results.uri?origin=searchhistory&shid=25) |
| **#10** | #8 AND NOT #9 | [2.009](https://www.scopus.com/search/history/results.uri?origin=searchhistory&shid=26) |
| **#11** | (KEY(infant* OR child*)) AND NOT (KEY(adult* OR aged)) | [2,357,005](https://www.scopus.com/search/history/results.uri?origin=searchhistory&shid=27) |
| **#12** | #10 AND NOT #11 | [1.699](https://www.scopus.com/search/history/results.uri?origin=searchhistory&shid=28) |
| **#13** | #12 AND ( LIMIT-TO(LANGUAGE,"English")) | [1.632](https://www.scopus.com/search/history/results.uri?origin=searchhistory&shid=30) |
| **#14** | #14 AND limit time 1970 - 2023 | [1.634](https://www.scopus.com/search/history/results.uri?origin=searchhistory&shid=34) |

**Table S3.** Search strategy performed in the systematic review and the number of publications identified in the Cochrane electronic database.

| **ID** | **Search in title and abstract** | **Trials and Reviews** | **Cochrane Reviews** |
| --- | --- | --- | --- |
| **#1** | whey | 2090 | 5 |
| **#2** | (((food OR macronutrient* OR eating) NEAR/6 (intake* OR habit* OR behavior* OR pattern*)) OR diet* OR intake OR ingestion OR suppl* OR consumption OR meal* OR nutrient* OR nutrit*) | 283971 | 4009 |
| **#3** | #1 AND #2 in Trials | 1886 | 5 |
